# Progress in Medicine

**Published:** 1897-03-13

**Authors:** 


### Progress in Medicine.

### FEVERS.

(*Continued from p.* 366.)

**Scarlatina.**—R. W. Marsden[1] gives a most interesting account of the joint affections, which, after Ashby's plan, he divides into (*a*) scarlatinal arthritis, (*b*) septic arthritis, (*c*) rheumatic synovitis, and (*d*) tubercular arthritis. The vast majority of these affections are not rheumatic at all. The true rheumatic form (*c*) is rarer even than the pyæmic; it occurs only in convalescence, or from the fifth to the eighth week; it is relieved by salicylates, and may affect the heart, and there is frequently a family history of rheumatism. On the other hand, the common type of joint trouble seems due to the specific poison, and generally appears from the sixth to the tenth day of the fever. It most often attacks the hands, forming a swelling along the extensor tendons on the dorsal surface, but the wrists and other joints may be affected. Marsden does not find that it is connected with ulceration of the throat or with rheumatism. It lacks the sweats, does not fly from joint to joint, and has few if any sequelæ. Though influenced by salicylates, their action on it differs from that seen in rheumatism. The septic type of arthritis occurs only in ·75 per cent. of scarlatina, and depends on severe throat affections. It may be acute or chronic, frequently goes on to suppuration, may occur at an early or late stage of the disease, and is very fatal. The tubercular is not only the rarest of the affections, but it is the latest in appearing, sometimes long after convalescence. H. G. Armstrong[2], in discussing the diagnosis of scarlatina from German measles, relies on the rule that the scarlatinal rash appears only on the forehead and not on the cheeks, while Rotheln covers the latter. In this he agrees very nearly with Goodall and Washbourn; but we must remember that the cheeks may show the ordinary flush of fever. The enlargement of the glands of the neck, though not occurring in measles, may appear in scarlatina as well as in Rotheln. Mammorek's serum has been used by Josias[3] in 145 cases. Where the serum was made by inoculating a sheep the mortality was only 2·08 per cent., but horse serum gave a death-rate nearly equal to that of the expectant plan, viz., 5·8 per cent. This serum has no effect on suppuration. Schill[4] reports on the use of the cold bath at a temperature of 95 deg. once or twice a day. A hundred and ten cases were thus treated, and only one developed severe symptoms. In the others there was no nephritis or other complications, which favours his view that the poison is eliminated by the skin. Ranke[5] found the diphtheria baccillus in more than half the cases of so-called scarlatinal diphtheria, and considers that scarlatina predisposes the patient to contract the other disease. He argues the desirability of using the diphtherial serum where these throats are seen, from the frequency with which the Loeffler bacillus is found in them. Another disease distinct from these affections is described at Fulham by Robert Lee.[6] It appears very like scarlatina, but is over in a week and desquamation is fine and branny, though the tonsils may ulcerate. It is often notified as being diphtheria. Five cases of scarlatina developing contemporaneously with enteric are detailed by Mcdowel Cosgrave, with notes of previous instances. It is important to notice, first, that each disease appears to exert a mitigating influence on the other; and, secondly, that a scarlatiniform rash is not uncommon in enteric, and is no evidence at all by itself of a double infection.

**Cholera.**—An inoculation against this disease was carried out on the line of the projected railway from Assam to Burmah at Margherita. The death-rate of the inoculated coolies only reached 2·5 per cent., while that of the others was nearly 19. The effect was so clear that all accepted treatment, and the epidemic entirely ceased.[7] The outbreak in Egypt practically came to an end in October, and throughout the year it was noticed that explosions of cholera could be traced to polluted water supplies.[8] During the Midnapore[9] attack in India, 46 wells were disinfected with permanganate of potash, and the outbreak ceased. An ounce or more was dissolved and added to the water; if the red tinge had vanished in half an hour, more was added. If too much was used frogs and fish were killed and the water became foul. It is not quite clear how it acts, since the solution is too weak to have a directly bactericidal action. Possibly it is by removing the food of the cholera microbe that it causes its rapid disappearance. Hankin[10] is careful to confine his claims for the value of the method within moderate limits. It is not put forward as a complete preventative of cholera or enteric, but it does reduce the number of the organisms in a water supply. Curiously enough, a natural antiseptic has been found existing in the water of the Jumna and the Ganges which destroys the cholera microbe very quickly. The substance has not been isolated, but it appears to be volatile, and is removed by boiling (unless this takes place in a sealed tube), or by the addition of alkaline peptone.

One of the most brilliant of Hankin's researches was the tracing of an outbreak of cholera to certain dish-cloths which had been washed in an infected stream at Sangor.[11] They were used for carrying a pot containing boiled water and one probably as a strainer for a chocolate jelly, which appears to be a remarkably good culture medium for cholera, so that Hankin found actually 9,000,000 of the microbes in a cubic centimetre of jelly made in the same way. In another place the typhoid microbe was found in a cistern though the source of the water was pure. The explanation proved to be that an old disused filter had been thrown in probably containing numerous germs, which were freely liberated in the cistern.

We noticed in the section on typhoid that much work had been done of late on the effects produced by the serum of immune animals on cultures of the organisms against which they were protected. Pfeiffer[12] showed that immunised animals have power to form specific bactericidal substances which destroy the one particular bacillus. When such bacilli are injected into the peritoneum they are quickly agglomerated, swell, and break up into granules. He does not believe the bactericidal substances are kept ready in the circulation, but are formed as required. Thus the effect of immunised serum, if added to a cholera culture and inserted into the peritoneum of a healthy animal, is quickly seen, but the process of disintegration stops in a drop of the mixture if it is placed in an incubator at body heat. That is, the serum causes the production in the animal of a bactericidal substance. Indeed, the bactericidal substances are distinct from the protective ones, which are more stable, but both exert their powers against specific bacilli only.

**The Plague.**—Hopes that were entertained that the Bombay outbreak might be due to some other disease proved illusory after the finding of Kitasato's microbe. It was first officially reported in Bombay on September 23rd, but the rats died in great numbers before the arrival of the plague was known,[13] and whether the infection was brought from Hong Kong or Bagdad is not clear. High fever, buboes in the groin or the axilla, with great prostration, followed by death in two or three days in 95 per cent. of the attacks, are the usual symptoms. Previous outbreaks in the district occurred in 1815, 1819, 1832, and 1857; while up country it has been found so late as 1893. A medical committee has been appointed by the Government to investigate the whole subject,[14] but the sudden outbreak and the public panic, which led to an enormous exodus of the inhabitants, have greatly interfered with the attempt to stop its ravages. Karachi was soon after declared infected, and it was stated that the plague was also found in Calcutta, though in a very mild form. The Bombay statistics gave 1,735 deaths out of 2,437 cases up to the 30th, but a great number were returned under the names of other diseases.[15] The Europeans were rarely attacked. Cantlie delivered a lecture on the disease before the Epidemiological Society.[16] He remarked that the distribution of the plague in the present century extended from the Mediterranean basin across Asia through the same latitudes to the shores of the Pacific. The rat, above all other animals, is affected by it, but pigs, dogs, and snakes are susceptible. The herbivorous animals and the cat never suffer from it, and where the bandicoot in Southern India replaces the common rat the plague is unknown. A mild fever, with glandular enlargement showing probably the same bacillus, is not uncommon in plague countries, and is possibly an attenuated form of the same disease, but rats do not die during an epidemic of this kind. In true plague we meet with the fulminant and typical varieties; the incubation period is usually three to six days; the infection is conveyed by dust or by prolonged and close association with the patients. The permanent home of the plague at the present time is Mesopotamia, whence it spreads at intervals to the surrounding countries, as was noticed above, from the Mediterranean to the Pacific. It is curious that the Chinese and Japanese suffer more from it than other races do, while Europeans are but little affected, their death-rate, when attacked, being only 20 per cent. Very few in Bombay have fallen victims, among whom unhappily was a prominent local physician.

**Malaria.**—Patrick Hehir[17] holds that there are two definite varieties of the parasite, the tertian and the quartan, though they are both present in the same patient in 14 per cent. of the cases. In the Deccan the quartan is the commonest, and it is the cause of the so-called "irregular" and "low" fevers, as well as of quartan and most quotidian agues. By the use of osmic acid vapour the organisms are fixed in the exact position and shape which they had in the living body. Afterwards they can be stained by methylene blue or Loeffler's alkaline stain. In some cases he examines the blood every half-hour from one attack to another. C. F. Craig[18] gives a useful account of the present state of our knowledge of these parasites, and recommends for staining a dilute solution of methylene blue in water mixed with an equal volume of a half per cent. solution of eosin in alcohol. The blood is dried on a slide, gently heated, and then placed on the stain for five minutes. It is then washed in water and mounted, the parasites are stained blue, and the corpuscles red. Thayer and Hewetson, who found the parasites in every one of 616 cases of malaria except two or three convalescents, believe that there is a third variety in America besides the two mentioned by Hehir; but no one has cultivated any form outside the body except Coronado, who claims to have obtained the organism from water. Bignami[19] reviews Manson's theory of the agency of mosquitoes in carrying the plasmodium, to which he is, on the whole, favourable. He refers to the difficulty that, if the organism passes a stage of its life in the mosquito, persons who go into non-malarial districts where mosquitoes exist would quickly infect these places, which has never, he thinks, been observed. Still, he collects a number of facts showing that the disease is probably conveyed to man by mosquitoes and similar insects. Whether it is normally conveyed from him, or can be, by them is another matter. Manson, in reply,[20] adduces the complexity, vigour, and long persistency of movement of the flagellæ as proof of their being alive, and refers to the infection of Mauritius and Reunion in recent days as an instance of the possible conveyance of the infection by man to mosquito-haunted but healthy countries. The outbreaks of malaria which occur when the soil is disturbed are against the universal conveyance of the germ to man by mosquitoes.

**Snake Poison.**—Calmette's request that his antivenomous serum should be tried effectively in English colonies has hardly yet been put to the test. At Meerut a boy[21] was bitten by a snake, described as a krait, a deadly species, but which was not killed. He was at once treated with the serum, and the wound washed with permanganate. Complete recovery followed. Near Cairo a girl was bitten by a snake, probably a cobra, which also escaped, and when she was nearly moribund the serum was injected. Very marked improvement and a complete recovery followed. Professor Ruffer and H. Keatinge[22] report this case.

[1] Med. Chronic., Aug. [2] B. Med. Journ., Dec. 26. [3] Amer. Journ. Med. Sciences, Oct. [4] B. Med. Journ., Jan. 16. [5] B. Med. Journ., Dec. 26. [6] B. Med. Jour., Dec. 12. [7] B. Med. Jour., Dec. 26. [8] B. Med. Jour., Oct. 31. [9] Amer. J. Med. Sciences, Nov. [10] B. Med. Jour.. Jan. 9, 16. [11] B. Med. Jour., Dec. 26. [12] Med. Chronic., Nov. [13] B. Med. Jour., Oct. 17, 24. [14] B. Med. Jour., Nov. 7. [15] B. Med. Jour., Jan. 2. [16] B. Med. Journ., Jan. 9. [17] Lancet, Dec. 5. [18] Med. Rec., Nov. 7. [19] Lancet, Nov. 14. [20] Lancet, Dec. 12. [21] B. Med. Jour., Nov. 21. [22] B. Med. Jour., Jan. 2.

